# Consistency of Safety and Efficacy of New Oral Anticoagulants across Subgroups of Patients with Atrial Fibrillation

**DOI:** 10.1371/journal.pone.0091398

**Published:** 2014-03-12

**Authors:** Jean-Christophe Lega, Laurent Bertoletti, Cyrielle Gremillet, Céline Chapelle, Patrick Mismetti, Michel Cucherat, Denis Vital-Durand, Silvy Laporte, on behalf of The Meta-Embol Group

**Affiliations:** 1 Département de Médecine Interne et Vasculaire, Centre Hospitalier Lyon Sud, Hospices Civils de Lyon, Université Claude Bernard Lyon 1, Lyon, France; 2 Groupe de Recherche sur la Thrombose, EA3065, Université de Saint-Etienne, Jean Monnet, Saint-Etienne, France; 3 Département de Médecine Thérapeutique, Centre hospitalo-universitaire de Saint-Etienne, Hôpital Nord, Saint-Etienne, France; 4 Inserm, CIE3, Saint-Etienne, France; 5 UMR CNRS 5558 Evaluation et Modélisation des Effets Thérapeutiques, Université Claude Bernard Lyon 1, Lyon, France; 6 Unité de Recherche Clinique, Innovation, Pharmacologie, Centre hospitalo-universitaire de Saint-Etienne, France; Loyola University Chicago, United States of America

## Abstract

**Aims:**

The well-known limitations of vitamin K antagonists (VKA) led to development of new oral anticoagulants (NOAC) in non-valvular atrial fibrillation (NVAF). The aim of this meta-analysis was to determine the consistency of treatment effects of NOAC irrespective of age, comorbidities, or prior VKA exposure.

**Methods and Results:**

All randomized, controlled phase III trials comparing NOAC to VKA up to October 2012 were eligible provided their results (stroke/systemic embolism (SSE) and major bleeding (MB)) were reported according to age (≤ or >75 years), renal function, CHADS2 score, presence of diabetes mellitus or heart failure, prior VKA use or previous cerebrovascular events. Interactions were considered significant at p <0.05. Three studies (50,578 patients) were included, respectively evaluating apixaban, rivaroxaban, and dabigatran versus warfarin. A trend towards interaction with heart failure (p = 0.08) was observed with respect to SSE reduction, this being greater in patients not presenting heart failure (RR = 0.76 [0.67–0.86]) than in those with heart failure (RR = 0.90 [0.78–1.04]); Significant interaction (p = 0.01) with CHADS2 score was observed, NOAC achieving a greater reduction in bleeding risk in patients with a score of 0–1 (RR 0.67 CI 0.57–0.79) than in those with a score ≥2 (RR 0.85 CI 0.74–0.98). Comparison of MB in patients with (RR 0.97 CI 0.79–1.18) and without (RR 0.76 CI 0.65–0.88) diabetes mellitus showed a similar trend (p = 0.06). No other interactions were found. All subgroups derived benefit from NOA in terms of SSE or MB reduction.

**Conclusions:**

NOAC appeared to be more effective and safer than VKA in reducing SSE or MB irrespective of patient comorbidities. Thromboembolism risk, evaluated by CHADS2 score and, to a lesser extent, diabetes mellitus modified the treatment effects of NOAC without complete loss of benefit with respect to MB reduction.

## Introduction

Non-valvular atrial fibrillation (NVAF) is a major cause of ischemic stroke and systemic embolism and is consequently characterized by increased mortality and morbidity and higher costs of medical care [1], [2]. Vitamin K antagonists (VKA), principally warfarin, have proved to be highly effective in preventing thromboembolic events in patients with paroxysmal, persistent, or permanent NVAF [3]. In 29 randomized trials involving more than 28,000 patients, pooled according to meta-analytic methods, adjusted-dose warfarin reduced the risk of stroke by 64% compared to the control and by 37% compared to aspirin, but at the cost of an increased risk of bleeding [3]. Furthermore, warfarin was associated with a 26% reduction in all-cause mortality, compared to no anticoagulation therapy, in randomized, controlled trials in patients with NVAF [3].

New oral anticoagulants (NOAC), directly inhibiting thrombin or factor Xa, have recently been developed. Their wide therapeutic windows allow the use of fixed doses without any need for laboratory monitoring [4], [5]. These new drugs could potentially overcome the well-known limitations of VKA, such as slow onset of action, need for regular blood sampling to monitor the international normalized ratio (INR), narrow therapeutic windows, marked inter-individual variations in drug metabolism, and multiple drug-drug and drug-food interactions, all of which lead to an increased risk of bleeding [6], [7], [8]. NOAC are associated with a reduced risk of stroke and systemic embolism as well as major bleeding, especially intracranial bleeding [9], [10], [11].

However, certain characteristics of patients with NVAF may modify the treatment effects of NOAC [12]. *Post hoc* analyses of trial data suggest that VKA-naïve patients have a different response when first treated with warfarin compared to those previously exposed to VKA, manifested by an increase in major bleeding [13], [14], [15]. Moreover, an age >75 years, comorbidities such as congestive heart failure, hypertension, diabetes mellitus, and previous stroke or transient ischemic attack, independently predict thromboembolism and are included in the CHADS2 score, the most reliably validated index for discriminating patients at higher risk of stroke [16]. Several of these factors, namely advanced age, previous stroke and hypertension, are also associated with a risk of bleeding, as assessed by the HAS-BLED score [17], [18]. These comorbidities consequently affect the incidence of thromboembolic or bleeding events, or both, and may modify the benefits and harms of NOAC. Apart from the interrelationship between risk factors for stroke and bleeding, and the issue of VKA status (prior exposure or no prior exposure), the interpretation of subgroup analyses corresponding to these comorbidities is hampered in published trials by the small number of outcome events within each subgroup and the lack of power to detect interactions. At the same time, the multiple interaction tests performed in each trial engendered a risk of type 1 error, i.e. a false positive conclusion in favor of superiority of the treatment investigated over the comparator. The aim of the present meta-analysis was to evaluate the consistency of the reductions in stroke and bleeding risks in patients with NVAF irrespective of their comorbidities and VKA status.

## Methods

### Inclusion criteria

The meta-analysis was performed according to a prospectively developed protocol (available from the corresponding author on request), which pre-specified the research objective, search strategy, study eligibility criteria, and methods of data extraction and statistical analysis. All subgroup variables were defined before the analyses.

Studies were eligible for inclusion in the present meta-analysis if they were randomized, controlled trials conducted in patients with NVAF and reported results according to CHADS2 score, age, presence of heart failure and diabetes mellitus, estimated glomerular filtration rate, prior exposure to VKA, and previous stroke or transient ischemic attack (TIA). Patients in the control group had to have received VKA and patients in the treated group had to have received an oral Factor Xa or thrombin inhibitor. Double-blind and open-label trial designs with or without blinded outcome evaluation were eligible.

### Data sources and searches

Medline (PubMed) and Embase were searched up to October 2012 using sensitive methods and employing the key words rivaroxaban, apixaban, betrixaban, edoxaban (DU-176b), eribaxaban, ximelagatran, dabigatran, LY 517717, darexaban (YM150), letaxaban, AZD0837, TTP889, RB006, MCC977 and TAK442 [19], [20]. Search terms included combinations of free text and medical subject headings (MeSH or Emtree). The complete search strategies may be requested from the authors. The references cited by the studies, reviews and meta-analyses retrieved by searching PubMed and Embase were also examined. Unpublished and ongoing trials were sought in clinical trial registers, including those of the National Institute of Health, the National Research Register, Current Controlled Trials, Meta-Embol and Trials Central. We also searched the Internet using the keywords listed above, including websites dedicated to the dissemination of clinical trial results, such as TheHeart.org, and the websites of the European Medicines Agency and the US Food and Drug Administration.

Unpublished studies were included in the meta-analysis if their design had been previously published in detail and patient characteristics, follow-up and the main results had been presented at international congresses. No restrictions concerning non-English language or small population size were applied. All qualifying studies were assessed for adequate blinding of randomization, completeness of follow-up, and objectivity of the outcome assessment. Phase II trials and studies with short-term follow-ups (<12 weeks) were excluded.

### Outcomes

The primary efficacy endpoint was the composite of stroke and systemic embolism. The primary safety endpoint was major bleeding (including both intracranial and extracranial bleeding), as defined by International Society on Thrombosis and Haemostasis [21].

### Data extraction

Studies were selected and data extracted by two reviewers (JCL and CC) independently. The risk of bias was assessed by the Cochrane Collaboration's tool. The hazard ratio (or the relative risk) and its confidence interval were extracted for all subgroups and directly included in the pooled results [22]. Data regarding inclusion criteria, events by subgroup and treatment were abstracted for each individual study or *post hoc* analysis. The results obtained on the intention-to-treat population were used for the main efficacy analyses. The risk of bias was assessed by the Cochrane Collaboration's tool [23]. Disagreements were resolved by a third reviewer. If a trial compared two NOAC treatments to the reference treatment (VKA), the number of patients in the reference arm was divided by two so that each patient was included in the meta-analysis only once.

### Statistical analysis

The relative risks (RR) or hazard ratios were weighted by the inverse of their variance and combined using the logarithm of RR method according to fixed-effect and random-effect models by R [24], [25]. Interaction was systematically tested for all subgroups and was considered as significant at p <0.05. The statistical heterogeneity between studies was assessed using Cochran's χ^2^ and I^2^ tests with a threshold of 0.10 [26]. In the event of heterogeneity, the results were pooled according to a random-effect model. Results were presented graphically, including the RR and corresponding 95% confidence intervals (95% CI).

## Results

### Literature search and study selection

**Figure 1:**
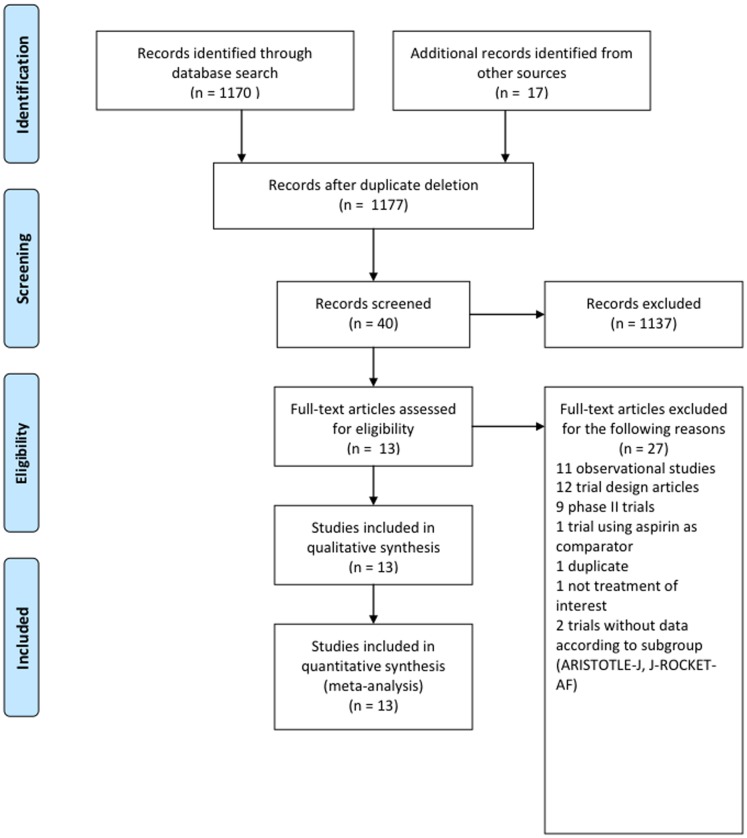
Flow chart for trial selection.

We identified 1170 references through electronic searches and 17 references by manual searches and contact with experts (Figure 1). Among these, three studies (including 50,578 patients) were eligible for analysis [9], [10], [11], the results of which were reported in 11 publications in peer-reviewed journals, one international congress abstract, and one Food and Drug Administration (FDA) report [27], [28], [29], [30], [31], [32], [33], [34], [35], [36]. Patient characteristics, study designs and methodological features are shown in Table 1. Patient inclusion criteria were based on various combinations of the known risk factors for thromboembolism included in the CHADS_2_ score and consequently the proportion of patients with a CHADS_2_ score <2 differed greatly from study to study, ranging from 0 to 34%. The proportion of VKA-naïve patients varied from 37% to 50%. The risk of bias according to the Cochrane Collaboration's tool mainly reflected the high quality of the trials included (Table 2). One prospective, randomized, open trial with a blind evaluation was included (Tables 1 and 2). Randomization was performed according to a computer-generated and centralized interactive voice-response system in all trials. One study stratified patients according to their prior VKA exposure and site of enrollment. In view of the few studies included, the source of heterogeneity was not explored.

**Table 1:** Characteristics of included studies.

Trial, year	Design	NOAC class	Mean age (years) Men (%)	VKA- naïve patients	Patients with CHADS2 score <2 (%)	ITT	Mean follow-up (months)	TTR (%)
RE-LY, 2009	Phase III PROBE	Thrombin inhibitor	71.64%	50%*	32%	All outcomes	24.0	64
ROCKET, 2011	Phase III Double-Blind	Anti- factor Xa†	73.60%	37%**	0%	Stroke and SE	23.2	55
ARISTOTLE, 2011	Phase III Double-Blind	Anti- factor Xa†	70.65%	43***	34%	All outcomes	21.6	62

ITT: intention-to-treat analysis; PROBE: prospective, randomized, open trial with a blind evaluation; SE, systemic embolism; TTR: time during which the INR was in the therapeutic range. † Doses were reduced in patients with renal failure. VKA-naïve was defined as * <63 and ***<31 days of lifetime VKA exposure, respectively, ** not defined in the trial protocol

**Table 2:** Assessment of the risk of bias according to the Cochrane Collaboration's tool.

RE-LY, 2009	+	+	−	+	+	+
ROCKET, 2011	+	+	+	+	+	+
ARISTOTLE, 2011	+	+	+	+	+	+
	Random sequence generation	Allocation concealment	Blinding of participants and personnel	Blinding of outcome assessment	Incomplete outcome data	Selective reporting

+: Low risk of bias; −: high risk of bias

Direct thrombin inhibitors were assessed in one study and direct factor Xa inhibitors in two studies (Table 1). Two trials used a reduced dose of the NOAC in patients with renal failure (apixaban 2.5 mg bid and rivaroxaban 15 mg, respectively) [9], [10], [37]. All studies used adjusted-dose warfarin (target INR, 2.0 to 3.0) as the comparator. The proportion of time during which the INR was in the therapeutic range ranged from 55% to 64% (Table 1). Renal function (GFR) was estimated by the Cockcroft-Gault method in all studies. The data according to subgroup are presented in the Table 3.

**Table 3:** Results of ARISTOTLE, RE-LY, and ROCKET-AF trials according to subgroup.

Trial Name, Year	Group	Outcome	Heart failure (Yes/No)	Previous stroke-TIA (Yes/No)	CrCl (<50 mL/min 50–80 mL/min >80 mL/min)	CHADS_2_ score	Age (>75 years/<75 years)	Diabetes mellitus (Yes/No)	Prior VKA useNaïve VKA patients
RELY, 2009	D 110 mg bid	SSE	HR 0.99 (0.69–1.42) HR 0.86 (0.67–1.09)	55/1195 128/4819	HR 0.89 (0.61–1.31) HR 0.91 (0.68–1.20) HR 0.83 (0.52–1.32)	0–1 42/19582 59/2088 >2 82/1968	HR 0.88 (0.66–1.17) HR 0.93 (0.70–1.22)	HR 0.74 (0.51–1.08) HR 0.97 (0.76–1.23)	94/3011 89/3004
		MB	HR 0.83 (0.64–1.09) HR 0.79 (0.67–0.94)	65/1195 277/4819	120/1151 154/2714 57/1899	0–1 74/1958 2 121/2088 >2 147/1968	204/2349 138/3666	96/1177 246/4837	166/3011 176/3004
	D 150 mg bid	SSE	HR 0.75 (0.51–1.10) HR 0.61 (0.47–0.79)	51/1233 83/4843	HR 0.47 (0.30–0.74) HR 0.65 (0.47–0.88) HR 0.71 (0.44–1.15)	0–1 26/1958 2 35/2137 >2 73/1981	HR 0.67 (0.49–0.90) HR 0.63 (0.46–0.86)	HR 0.62 (0.42–0.91) HR 0.66 (0.51–0.88)	73/3049 61/3026
		MB	HR 0.79 (0.60–1.03) HR 0.99 (0.84–1.16)	102/1233 297/4843	123/1188 182/2777 80/1882	0–1 84/1958 2 127/2137 >2 188/1981	246/2466 153/3610	117/1124 282/4952	209/3049 190/3026
	VKA	SSE	-	65/1195 137/4827	-	0–1 40/1859 2 60/2230 >2 102/1933	-	-	105/2929 97/3093
		MB	-	97/1195 324/4827	112/1081 206/2806 94/1887	0–1 105/1859 2 144/2230 >2 172/1933	206/2423 215/3599	102/1195 319/4827	216/2929 205/3093
	R 15 or 20 mg od	SSE	160/4438 109/2642	187/3892 82/3189	77/1490 126/3298 65/2285	2 30/924 >2 239/6156	125/3082 144/3999	95/2851 174/4230	168/4413 101/2668
ROCKET, 2010		MB	106/4428 83/2632	136/3881 53/3180	50/1485 91/3290 47/2278	2 21/922 >2 168/6138	82/3073 107/3988	70/2842 119/4219	114/4401 75/2660
	VKA	SSE	172/4413 134/2676	190/3875 116/3215	86/1459 151/3400 68/2222	2 36/933 >2 270/6155	154/3082 152/4008	114/2796 192/4294	175/4440 131/2650
		MB	141/4409102/2672	151/386992/3213	60/1456 128/3396 54/2221	2 24/931>2 219/6149	124/3077119/4005	94/2793149/4289	140/4437103/2645
	A 2.5 or 5 mg bid	SSE	70/3235 142/5885	73/1748 139/7372	54/1502 87/3817 70/3761	0–1 44/3100 2 74/3262 >2 94/2758	79/2850 133/6270	57/2284 155/6836	102/5208 110/3912
ARISTOTLE, 2011		MB	87/3235 240/5885	77/1748 250/7372	73/1502 157/3817 96/3761	0–1 76/3100 2 125/3262 >2 126/2758	151/2850 176/6270	112/2284 215/6836	185/5208 142/3912
	VKA	SSE	79/3216 186/5865	98/1790 167/7291	69/1515 116/3770 79/3757	0–1 51/3083 2 82/3254 >2 132/2744	109/2828 156/6253	75/2263 190/6818	138/5193 127/3888
		MB	137/3216 325/5865	106/1790 356/7291	142/1515 199/3770 119/3757	0–1 126/3083 2 163/3254 >2 173/2744	224/2828 238/6253	114/2263 348/6818	274/5193 188/3888

A: apixaban; Bid: twice daily; D: dabigatran; HR: hazard ratio; MB: major bleeding; NR: not reported; od: once daily; R: rivaroxaban; SE: systemic embolism; SSE: stroke and/or systemic embolism; TIA: transient ischemic attack; VKA: vitamin K antagonist.

### Stroke/systemic embolism and major bleeding risk reduction

#### Age and renal insufficiency

**Figure 2:**
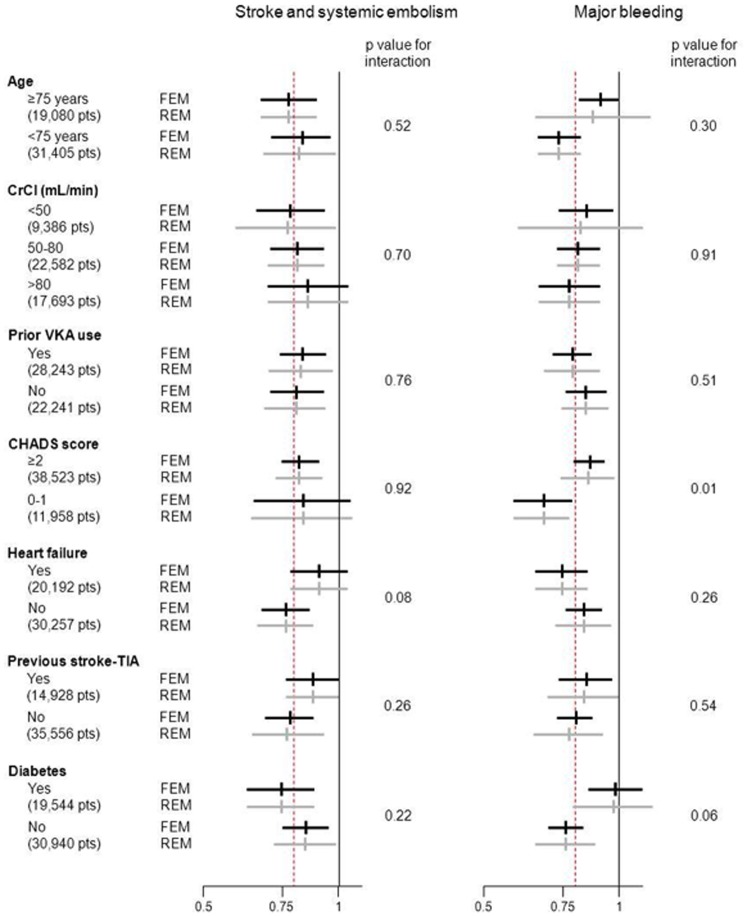
Relative risk of stroke and systemic embolism and major bleeding reduction according to age, comorbidities and VKA status.

**Figure 3:**
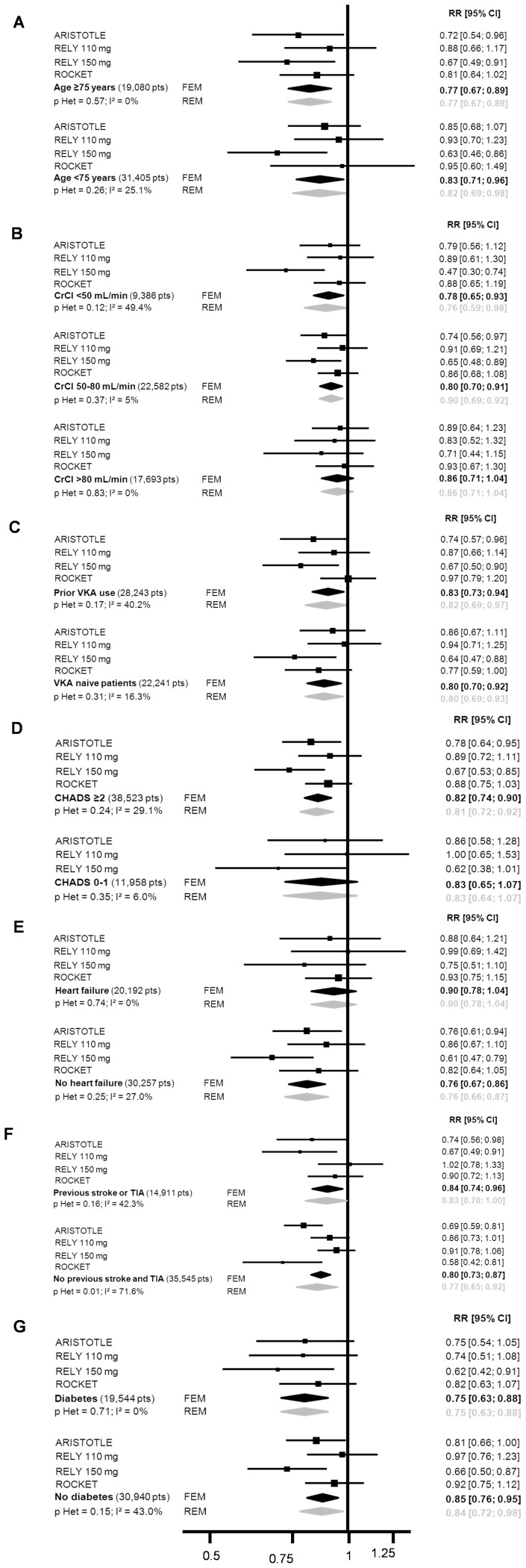
Detailed forest plot of stroke and systemic embolism according to (A) age, (B) renal function, (C) prior VKA exposure, (D) CHADS_2_ score, (E) heart failure, (F) prior stroke or transient ischemic attack, (G) diabetes mellitus.

**Figure 4:**
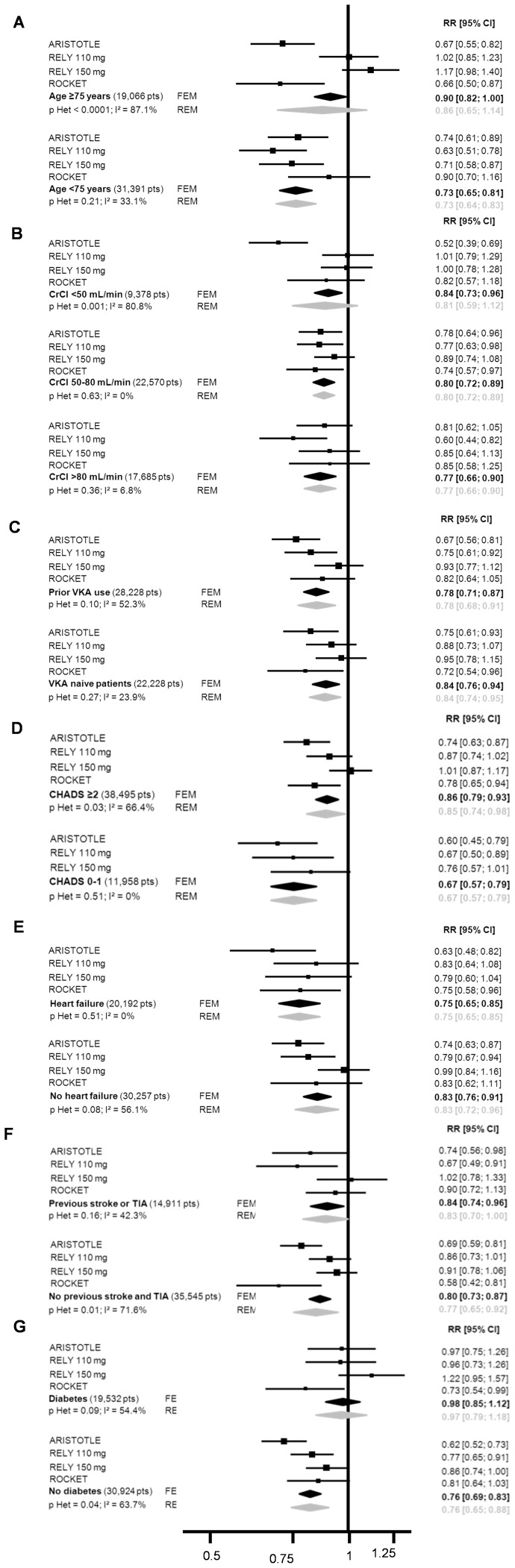
Detailed forest plot of major bleeding according to (A) age, (B) renal function, (C) prior VKA exposure, (D) CHADS_2_ score, (E) heart failure, (F) prior stroke or transient ischemic attack, (G) diabetes mellitus.

Treatment benefit with regard to SSE risk reduction favored NOAC compared to VKA in both patients aged over 75 years and younger patients (Figures 2 and 3). The benefit remained in favor of NOAC with regard to major bleeding, even though the reduction in risk was lower in elderly patients (RR = 0.86 [0.65–1.14]) than in younger patients (RR = 0.73 [0.64–0.83], p interaction = 0.30) (Figures 2 and 4). Similar results were observed in patients with normal renal function and those with moderate or severe renal impairment, the reductions in SSE and MB being similar in the two subgroups. No interaction was found between these two subgroups.

#### Previous exposure to VKA

NOAC were superior to VKA irrespective of subgroup, reductions in SSE (Figure S2 and S3) and MB (Figures 2 and 4) being seen in both VKA-naïve patients (RR = 0.80 [0.70–0.92] and RR = 0.84 [0.76–0.94], respectively) and those previously exposed to VKA (RR = 0.83 [0.73–0.94] and RR = 0.78 [0.68–0.91], respectively), with no interaction.

#### CHADS_2_ score

The treatment effect of NOAC in terms of SSE reduction was similar (p =  0.92) irrespective of the CHADS_2_ score (Figures 2 and 3). The risk of thromboembolism, as defined by the CHADS_2_ score, significantly modified the effect of NOAC on MB reduction (p interaction = 0.01), a greater effect being evident in patients with CHADS_2_ scores of 0–1 (RR = 0.67 [0.57–0.79]) than in those with CHADS_2_ scores ≥2 (RR = 0.85 [0.74–0.98]) (Figures 2 and 4).

#### Comorbidities included in the CHADS_2_ score: heart failure, prior stroke/transient ischemic attack, and diabetes mellitus

A trend towards interaction with heart failure (p = 0.08) was observed with respect to SSE reduction, this being greater in patients not presenting heart failure (RR = 0.76 [0.67–0.86]) than in those with heart failure (RR = 0.90 [0.78–1.04]) (Figures 2 and 3). As regards MB, the test for interaction showed a non-significant trend (p = 0.06) towards a difference between patients with (RR = 0.97 [0.79–1.18]) and without (RR = 0.76 [0.65–0.88]) diabetes mellitus (Figures 2 and 4). No interaction was detected for any other comorbidity considered.

## Discussion

The goal of this study was to assess the consistency of the benefit-risk balance of NOAC in patients with NVAF irrespective of their characteristics. Overall, our meta-analysis showed a similar treatment effect of NOAC in almost all the subgroups encountered in clinical practice, with no qualitative interaction in terms of SSE or MB reduction, i.e. no reversal of treatment effect leading to an increase of events with NOAC compared to warfarin. However, there was a significant quantitative interaction, expressed by a difference in magnitude of the treatment effect according to subgroup, the effect of NOAC with regard to MB reduction being smaller in patients with a high risk of SSE (CHADS_2_ score ≥2). There was also a strong trend towards interaction with diabetes mellitus in patients with a CHADS_2_ score ≥2. It is conceivable that co-prescription of antiplatelet drugs, more frequent in patients with a CHADS_2_ score ≥2 or diabetes mellitus, might explain an increased incidence of bleeding events but *post hoc* analysis of the RE-LY trial did not indicate an interaction with co-administration of clopidogrel or aspirin in terms of MB [38]. Some authors have questioned the repercussions of the variable proportion of patients with a high CHADS2 score across phase III trials [39], [40]. In particular, the population included in the ROCKET-AF trial differed from those of ARISTOTLE and RE-LY in that it comprised a higher proportion of patients with comorbidities. In addition, heart failure may modify the benefit of NOACs with respect to SEE reduction, but the magnitude of the interaction did not permit to draw firm conclusions. Our results tend to corroborate this concern and call for careful interpretation of indirect comparisons of the results of trials assessing NOAC [40].

In published trials, the safety and efficacy profiles of NOAC were not worse than those of VKA, irrespective of patient age and prior exposure to VKA [15], [32]. Moreover, all subgroups derived a significant benefit from these new drugs in terms of reductions in MB and/or SSE. NOAC reduced major bleeding in all subgroups at risk of this iatrogenic event, such as those aged ≥75 years, those having experienced a stroke in the past, those with a high CHADS2 score and those presenting renal impairment [18], [31]. The two subgroups at greatest risk of NOAC accumulation, i.e. elderly patients and those with renal failure, both showed a higher incidence of bleeding and thromboembolic events [41]. However, both these subgroups nevertheless derived benefit from NOAC in terms of diminished SSE risk, with no signal indicating an increase in MB, except in the case of dabigatran 150 mg, which was associated with a trend towards an increased risk of MB compared to VKA. In addition, comparison of both dabigratran doses with warfarin revealed a significant statistical interaction between treatment and risk of major bleeds in elderly patients [34]. In patients with renal failure, subgroup analysis showed a heterogeneity of treatment effect, related to a relative increase in bleeding events with dabigatran compared to rivaroxaban and apixaban. We postulated that the percentage renal clearances of 80%, 33% and 25% respectively, in the three treatment groups, might have led to an increased bleeding risk with dabigatran due to drug accumulation. Overall, the reduction in the rate of SSE observed with NOAC versus VKA was similar in patients at increased risk of thromboembolism events, such as those having experienced a prior TIA/stroke, those presenting diabetes mellitus or heart failure, and those aged ≥75 years [42]. The results were same whether or not the patients had previously been exposed to VKA.

Our study suffers from several limitations. First, it comprised a meta-analysis of subgroups. However, most of these subgroups were well defined and included in the stratification scheme for randomization in each study. As discussed above, it is likely that the the studies included in this meta-analysis were not powered to reach significance for many outcomes in subgroups such as those comprising patients with a CHADS2 score <2, those having previously experienced a TIA or stroke or those with a GFR<50 mL/min, due to the small population sizes. Second, we found significant heterogeneity for eight subgroups, but unfortunately, could not analyze its possible causes in view of the small number of trials included. Heterogeneity was mainly observed with respect to MB, a composite outcome encompassing both intracranial and extracranial bleeding. Whereas the disparity between the effect of NOAC and that of VKA followed the same trend in all trials with respect to SSE, the results for MB diverged, the risk of gastrointestinal bleeding being greater with rivaroxaban and with dabigatran at 150 mg than with VKA [9], [11], [43]. Besides the interaction between CHADS2 score and treatment effect, the intrinsic pharmacodynamic properties of the different drugs, e.g. their extent of renal excretion, might explain such differences in the reduction of extracranial bleeding. Finally, we could not exclude inflation of the type 1 error due to the multiple tests performed. For this reason, we choose a conservative threshold of significance (p <0.05) to limit the risk of false positive results despite the lack of power of the interaction test [44].

In conclusion, NOAC appear to be more effective and safer than VKA in reducing SSE or MB irrespective of patient comorbidities. The risk of thromboembolism, as evaluated by the CHADS2 score, and to a lesser extent the presence of diabetes mellitus and heart failure, modified the treatment effect of NOA without complete loss of benefit in terms of MB reduction. Other comorbidities, especially moderate renal impairment or prior VKA use, were not associated with significant differences in treatment effect with regard to either bleeding or ischemic risk reduction. Overall, these new drugs were beneficial for all patient subgroups in the absence of any contraindication.

## Supporting Information

Checklist S1 (DOC)
